# Trend in the epidemiological risk of leprosy in the state of Goiás-Brazil between 2010 and 2021

**DOI:** 10.1590/S2237-96222024v33e20231435.en

**Published:** 2024-08-23

**Authors:** Caio Cesar Barbosa, Rafael Alves Guimarães, Nayara Figueiredo Vieira

**Affiliations:** 1 Universidade Federal de Goiás, Faculdade de Enfermagem, Goiânia, GO, Brazil

**Keywords:** Estudios de Series Temporales, Epidemiología, Vigilancia Epidemiológica, Vigilancia en Salud, Lepra, Time Series Studies, Epidemiology, Epidemiological Monitoring, Health Surveillance, Leprosy

## Abstract

**Objective:**

To analyze trends in epidemiological risk of leprosy in Goiás state, Brazil, and its health macro-regions, between 2010 and 2021.

**Method:**

This is a time series analysis of the composite leprosy epidemiological risk index in Goiás. We used cases held on the Notifiable Health Conditions Information System for calculating indicators separately and risk, classified as high, medium, low and very low. Trends were analyzed using Prais-Winsten linear regression and risk maps were produced.

**Results:**

Goiás showed high leprosy endemicity (24.8 cases/100,000 inhabitants) and medium epidemiological risk between 2019 and 2021 (0.58). A stationary trend was found (annual percentage change, 0.50; 95% confidence interval, -3.04; 4.16) for risk of leprosy in Goiás as a whole and in its Central-West and Central-Southeast macro-regions.

**Conclusion:**

There is need for actions to reduce the epidemiological risk of leprosy, especially where its trend is stationary, this includes early screening for new cases and health education.

## Study contributions

**Main results:**

Leprosy persists in Goiás state, Brazil, in an endemic form, with heterogeneous distribution. There has been a reduction in the number of municipalities with high epidemiological risk of leprosy, but challenges include active transmission and late diagnosis.

**Implications for services:**

Long-term strategies for prevention, early detection, treatment and monitoring of people with leprosy and their contacts are needed.

**Perspectives:**

It is crucial to strengthen health policies targeting leprosy in Goiás state, prioritizing continuing education and training programs for health professionals working in the entire territory.

## RESUMEN

**Objetivo:**

Evaluar la tendencia de riesgo epidemiológico de lepra en el estado de Goiás y macrorregiones sanitarias de 2010 a 2021.

**Método:**

Análisis de serie temporal del indicador compuesto del índice de riesgo epidemiológico de lepra en Goiás. Se utilizaron los casos del Sistema de Enfermedades de Declaración Obligatoria para calcular indicadores aislados y se clasificó el riesgo en alto, medio, bajo y muy bajo. La tendencia se analizó mediante regresión lineal de Prais-Winsten y se elaboraron mapas de riesgo.

**Resultados:**

Goiás presentó alta endemicidad de lepra (24,8 casos/100 mil habitantes) y riesgo epidemiológico medio en 2019 y 2021 (0,58). Se observó una tendencia estacionaria (variación porcentual anual, 0,50; intervalo de confianza, -3,04; 4,16) del riesgo de lepra en Goiás y en las macrorregiones Centro-Oeste y Centro-Sureste.

**Conclusión:**

Es necesario implementar acciones para reducir el riesgo epidemiológico de lepra, especialmente con una tendencia estacionaria, y esto incluye medidas de rastreo y diagnóstico precoz de nuevos casos y educación sanitaria.

## INTRODUCTION

Leprosy continues to be a health problem in several countries, including Brazil.^1,2^ A total of 174,087 new cases of the disease were estimated to have occurred in 2022, with an increase of 23.8% in relation to 2021.^1^

Brazil ranks second worldwide in new cases of leprosy in absolute terms.^1^ The detection rate of new leprosy cases in Brazil was 8.58 cases/100,000 inhabitants, indicating average endemicity,^3^ and the need to implement actions to achieve the goals established by the Ministry of Health National Strategy for Combating Leprosy 2024-2030.^4^

The leprosy case detection rate showed a falling trend in Brazil from 2011 to 2022, mainly due to multidrug therapy and strategic actions of the Brazilian National Health System, such as decentralization of leprosy control actions to primary health care. However, there was an accelerated reduction, greater than expected, during the covid-19 pandemic, between 2020 and 2022.^3^ The public health measures adopted to address covid-19, such as isolation and restrictions on circulation, made it difficult to carry out population-based actions necessary for early detection of new cases, such as active searching, testing contacts and other examinations. Moreover, there was a reduction in access to leprosy services in the same period.^5^

Studies conducted in Brazil and China^6-10^ have evaluated the temporal trend of separate indicators, with relevant assessment of epidemiological risk in a comprehensive manner. Using the composite indicator of epidemiological risk of leprosy, the Brazilian states of Paraíba^11^ and Minas Gerais^12^ analyzed spatial distribution of risk, revealing unequal distribution of the epidemiological risk of leprosy among the municipalities that comprise those states.

The state of Goiás stands out in Brazil in terms of its high leprosy detection rates and areas at risk of the disease.^3^ A falling trend was seen in new case detection between 2007 and 2017, a pattern similar to that found in other Brazilian regions. High proportions of physical disability^13^ and cases in children reflect late diagnosis and recent transmission, which reinforces the need for coordinated strategies to reduce its impact and for studies on leprosy case distribution.^13,14^

Analysis of the dynamics of the spatial distribution of health problems and diseases and the trend of epidemiological risk, especially in the case of leprosy, in endemic regions, such as the state of Goiás, is beneficial for identifying risk according to geographic space. Furthermore, this study can contribute to monitoring the effectiveness of the actions of the National Strategy for Combating Leprosy 2024-2030.^4^ The objective of this study was therefore to analyze the trend of epidemiological risk of leprosy in the state of Goiás, Brazil, and its health macro-regions between, 2010 and 2021. 

## METHODS

This is a times series study of trend of epidemiological risk of leprosy in Goiás from 2010 to 2021. 

The study was performed based on data from January 1^st^ 2010 to December 31^st^ 2021. The unit of analysis was the state of Goiás and its five health macro-regions, namely: Central-North, Central-West, Central-Southeast, Northeast and Southwest. The state is located in the Midwest region of Brazil and has epidemiological importance on the national scenario. 

The study population was comprised of new leprosy cases resident in the state of Goiás notified between 2010 and 2021. 

The data were extracted from the Notifiable Health Conditions Information System, having been made available by the Goiás State Health Department. The cases were filtered according to the “new case” entry mode, and those with an outcome recorded as “diagnostic error” were excluded. 

In order to build the composite indicator of the leprosy epidemiological risk index for Goiás, we used the annual detection rate of new leprosy cases per 100,000 inhabitants, the annual detection rate of new leprosy cases in the population up to 14 years of age per 100,000 inhabitants and the rate of new leprosy cases with grade 2 physical disability at diagnosis. The methodology followed Brazilian Ministry of Health recommendations.^15^

After calculating the indicators mentioned above, by year and municipality, a mathematical equation was applied to obtain a ratio between the observed value (average of values recorded annually) and the maximum value (highest value observed in the years assessed),^11^ by municipality, macro-region and the state as a whole. Following this, in order to calculate the leprosy epidemiological risk index, the three indicators were added together and divided by three. The average values varied between 0 and 1, with “best” being the lowest value (0) and “poorest” being the highest value (1).

The risk maps were built using the composite indicator of the leprosy epidemiological risk index by municipality and second three-year period of the time series. In accordance with previous studies,^11,12^ the values were classified based on distribution quartiles and classified as: very low risk (< 0.2), low risk (0.21-0.4), medium risk (0.41-0.6) and high risk (> 0.6). Subsequently, choropleth maps were created using RStudio software, version 1.4.1106.

The temporal trend analyses were performed using Stata statistical software, version 17.0 (StataCorp LP, College Station, United States). 

We used the Prais-Winsten linear regression model with robust variance and adjustment for Durbin-Watson autocorrelation to analyze the temporal trend of the leprosy epidemiological risk index in the state of Goiás and its macro-regions, between 2010 and 2021.^16^

The study’s dependent variable (Y) was the leprosy epidemiological risk index, while the independent variable was the year of the time series. Before inclusion in the regression models, the dependent variable (Y) was transformed into a base-10 logarithm. This procedure made it possible to minimize heterogeneity of variance in the residuals of the regression model and contributed to better calculation of the temporal trend.^16^ The Prais-Winsten regression equation was as follows:


$$Log(Y_{t})=\beta_{0}+\;\beta_{1}x\;+\;e_{t},$$

where: *Log* (*Y* *t*) was the study’s dependent variable, i.e. the leprosy epidemiological risk index after transformation into a base-10 logarithm, *β* 0 was the constant or intercept; *β*_1_ was the slope of the straight line or linear trend coefficient; *x* was the independent variable, i.e. the year of the study, and *e* *t* was the random error. The subscript “t” estimated the times of the data set {t_1_, ..., t_12_}, whereby t_1_ = 2010 and t_19_ = 2021. The *β*_1_ coefficients and robust standard errors were obtained though Prais-Winsten regression analysis, while annual percentage change (APC) and 95% confidence intervals (95%CI) were calculated using the following formulae:^16^


$$VPA=(1\;+\;10{)}^{\beta_{1}}*100,$$

where *β* 1 is the slope of the straight line or the linear trend coefficient 


$$IC95\%=(1\;+\;10^{\left(\beta_{1}\pm t*EPR\right)})*100,$$

where *β* 1 is the slope of the straight line or the linear trend coefficient, t is the value of Student’s t distribution and has 11 degrees of freedom (12 years of the time series – 1) at a two-tailed 95%CI and EPR is the robust standard error.

The t-statistic was used to establish the statistical significance of the models and p values < 0.05 were considered to be statistically significant. Based on the APC results and p-values obtained in the regression models, the trends were classified as rising (positive APC and significant p-value), falling (negative APC and significant p-value) or stationary (positive or negative APC and p-value not significant). The coefficient of determination (*R* ^2^) was used to check the regression model fit.

The study was approved by the Research Ethics Committee of the Hospital das Clínicas of the Universidade Federal de Goiás, as per Certificate of Submission for Ethical Appraisal No. 58175122.3.0000.5078 and Opinion No. 5.421.173, dated May 20, 2022.

## RESULTS

Between 2010 and 2021, 19,728 new leprosy cases were reported in Goiás, 814 (4.13%) in children under 15 years of age and 1,264 (6.40%) cases with grade 2 physical disability at diagnosis. 

Table 1 shows the averages of the indicators used to calculate the composite indicator, by macro-regions of the state of Goiás. The annual averages of the annual detection rates of new leprosy cases, annual detection rates of new leprosy cases in the population up to 14 years old and those of new leprosy cases with grade 2 physical disability at diagnosis in Goiás between 2010 and 2021 were 24.8, 4.5 and 1.5 cases per 100,000 inhabitants, respectively. The Central-West, Central-North and Central-Southeast macro-regions had a new leprosy case detection rate above the state average (Central-West: 39.0 cases/100,000 inhabitants, Central-North: 25.1 cases /100,000 inhabitants and Central-Southeast: 29.2 cases/100,000 inhabitants). Similarly, those macro-regions had a detection rate of new leprosy cases in the population up to 14 years of age that was higher than the state average (Central-West: 7.0 cases/100,000 inhabitants, Central-North: 4.3 cases/100,000 inhabitants and Central-Southeast: 6.2 cases/100,000 inhabitants). Finally, three regions also had a rate of new leprosy cases with grade 2 physical disability at diagnosis higher than the state average (Central-West: 2.6 cases/100,000 inhabitants, Central-Southeast: 2.0 cases/100,000 and Southwest: 1.6 cases/100,000 inhabitants).

**Table 1:** Annual detection rate of new leprosy cases in the general population and up to 14 years old and rate of new leprosy cases with grade 2 physical disability at diagnosis in the state of Goiás, Brazil, between 2010 and 2021 (n = 19,728)

Unit of analysis	Annual detection rate of new leprosy cases	Annual detection rate of new leprosy cases, in the population aged up to 14 years	Rate of new leprosy cases with grade 2 physical disability at the time of diagnosis
Central-North	39.0	7.0	2.6
Central-West	25.1	4.3	1.1
Central-Southeast	29.2	6.2	2.0
Northeast	11.4	2.6	0.9
Southwest	20.5	2.4	1.6
Goiás	24.8	4.5	1.5

**Figure 1:**
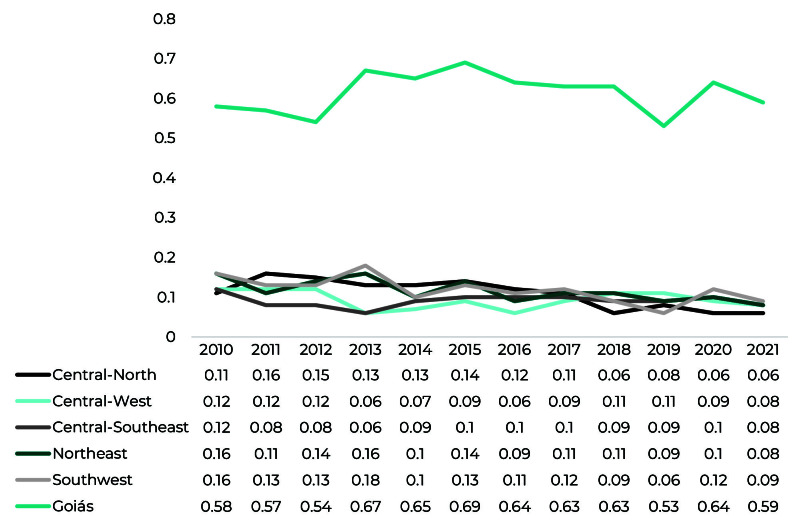
Leprosy epidemiological risk index by macro-region of the state of Goiás, Brazil, between 2010 and 2021 (n = 19,728)

In Goiás as a whole, there was an increase of 1.72% in the leprosy epidemiological risk index from 2010 to 2021 (0.58 to 0.59) (Figure 1). Percentage change was -45.45% in the Central-North (0.11 to 0.06), -33.33% in the Central-West (0.12 to 0.08), - 33.33% in the Central-Southeast (0.12 to 0.08), -50.00% in the Northeast (0.16 to 0.08), and -43.75% in the Southwest (0.16 to 0.09). 

The percentage of municipalities with a “very low” risk classification ranged from 24.4% to 45.1% between the 2010/2012 three-year period and the 2019/2021 three-year period, an increase of 84.83%. On the other hand, the percentage of municipalities classified as “high risk” varied from 11.0% to 3.7% between the 2010/2012 three-year period and the 2019/2021 three-year period, a reduction of 66.36%.

**Figure 2:**
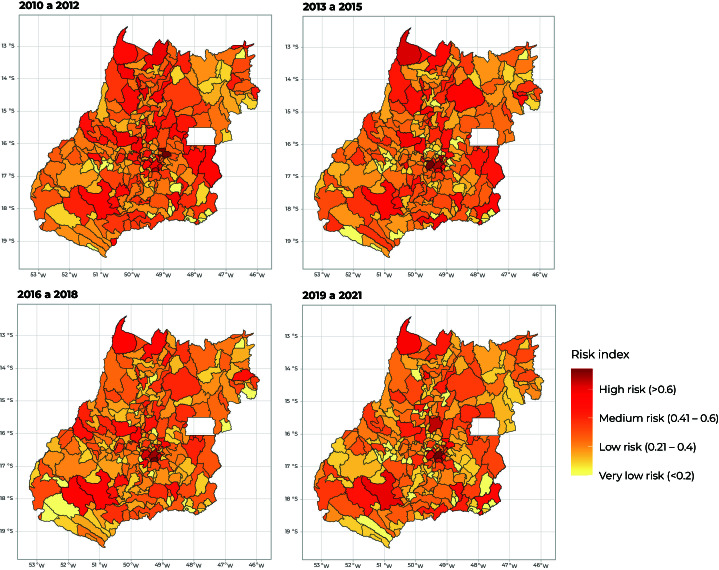
Spatial distribution by three-year period of epidemiological risk of leprosy per municipality of the state of Goiás, Brazil, between 2010 and 2021 (n = 246)

Figure 2 shows the distribution of the epidemiological risk of leprosy in the state’s municipalities by three-year period. The Central-North, Central-West and Central-Southeast health macro-regions recorded the highest number of municipalities classified as having high epidemiological risk of leprosy.

**Table 2:** Evolution of the classification of epidemiological risk of leprosy in the municipalities of the state of Goiás, Brazil, by three-year assessment period, between 2010 and 2021 (n = 246)

Risk classification	2010-2012		2013-2015		2016-2018		2019-2021	
	N	%	N	%	N	%	N	%
Very low	60	24.4	74	30.1	91	37.0	111	45.1
Low	115	46.7	109	44.3	107	43.5	103	41.9
Medium	44	17.9	41	16.7	38	15.4	23	9.3
High	27	11.0	22	8.9	10	4.1	9	3.7

The leprosy epidemiological risk index in the state of Goiás showed a stationary trend in the period under study (APC = 0.50; 95%CI -3.04;4.16). The stationary trend was also observed in the Central-West (APC = -4.04; 95%CI -12.69;5.46) and the Central-Southeast (APC = 0.17; 95%CI -13.21; 16.57) macro-regions. On the other hand, a falling trend was observed in the Central-North (APC = -17.69; 95%CI -24.40; -10.38), the Northeast (APC = -9.17; 95%CI -13 .21;-6.18) and the Southwest (APC = -11.82; 95%CI -18.01;-5.17) macro-regions (Table 3).

**Table 3:** Annual percentage change (APC), 95% confidence interval (95%CI) and coefficient of determination (R²) of the leprosy epidemiological risk index, per macro-region of the state of Goiás, Brazil, between 2010 and 2021

Unit of analysis	APC^a^ (95%CI)	p-value	R^2 b^	Trend
Central-North	-17.69 (-24.40;-10.38)	0.002	0.668	Falling
Central-West	-4.04 (-12.69;5.46)	0.359	0.001	Stationary
Central-Southeast	0.17 (-8.70;16.57)	0.969	0.001	Stationary
Northeast	-9.17 (-13.21;-6.18)	< 0.001	0.953	Falling
Southwest	-11.82 (-18.01;-5.17)	0.003	0.802	Falling
Goiás	0.50 (-3.04;4.16)	0.768	0.084	Stationary

a) APC: Annual percentage change; b) Prais-Winsten linear regression; R^2^: Coefficient of determination.

## DISCUSSION

The results of this study indicate that leprosy persists as a public health problem in the state of Goiás. The annual averages of the new leprosy case annual detection rates in the population up to 14 years old and those of new leprosy cases with grade 2 disability were high, especially in the Central-West, Central-North and Central-Southeast macro-regions. This suggests significant endemicity, recent transmission with the presence of cases in children and late diagnosis. The percentage of municipalities with high risk of leprosy reduced in Goiás from 2010/2021 to 2019/2021. However, heterogeneous distributions were noted in the number of municipalities with high risk of leprosy. The Central-North, Central-West and Central-Southeast health macro-regions were those that registered the highest number of municipalities classified as having high epidemiological risk of leprosy. Finally, a stationary trend was found for epidemiological risk of leprosy in the state of Goiás as a whole and a falling trend for three of its macro-regions (Central-North, Northeast and Southwest).

This study used secondary data sources, which may contain inconsistencies in information quality. On order to minimize biases or errors, the data were treated with ethical and methodological rigor. In the last three-year period assessed (2019-2021), there may have been undiagnosed cases of leprosy due to the covid-19 pandemic and, therefore, underestimation of the epidemiological situation of the disease. Therefore, new studies on epidemiological risk of leprosy are recommended for the period after 2020 so as to confirm the endemic situation in the state’s macro-regions. 

Hanseniasis persists as a problem in Goiás, especially in the Central-North macroregion, with high disease endemicity and late diagnosis. This macro-region of the state of Goiás has been historically marked by a higher number of cases, due to its location on the border with states which have high endemicity, such as Mato Grosso and Tocantins.^3^ As such, the migratory process may explain the disparity of the Central-North macro-region in relation to the state of Goiás as a whole. However, the disease may be associated with other factors, such as greater environmental or individual vulnerability.^17^

With an average of 1,685 cases per year between 2009 and 2022,^3^ the state of Goiás is far from the elimination target proposed by the Ministry of Health 2024-2030 Strategy, which foresees leprosy being eliminated following absence of autochthonous cases for three consecutive years after transmission interruption.^4^ Notwithstanding, Goiás has a falling trend in detection of new leprosy cases, this being the same scenario observed worldwide.^1,11,14^ We believe that the absolute number of new leprosy cases, proposed by the strategy, is not the best way to assess interruption of transmission, but that this would be better achieved using a set of indicators that allow robust assessment of the stage of leprosy elimination. 

However, different dynamics were found in the rate of cases with grade 2 physical disability, which has shown an increasing trend in published research.^9,13^ The persistence of cases diagnosed with grade 2 physical disability at high levels highlights late diagnosis and contributes to the formation of leprosy transmission foci.^11,18^ A study conducted in Ethiopia showed an average delay of 12 months in diagnosis, culminating 83% grade 2 physical disability among the individuals assessed.^19^

Three pillars are necessary for early leprosy detection: health education for the population, training of health professionals and investment in health policies that address the needs of the population. Providing health education to communities is vital to support early recognition of leprosy symptoms and reduce prejudice and stigmatization of people with leprosy and their families.^19^ These factors can contribute to reducing delays in diagnosis and preventing physical disabilities.

Since 2019, the number of leprosy cases in children under 15 years of age has been decreasing in the state of Goiás, however, an average of 67 child cases per year was found between 2009 and 2022.^20^ This demonstrates recent transmission of the disease, and also that there is a need to reflect on strategies to achieve the current Ministry of Health target, which plans for absence of autochthonous cases in people under 15 for a period of five consecutive years.^4^ A study conducted in China found a falling trend in new leprosy cases in children between 2011 and 2020, but the cases were grouped in high-risk areas, which were considered as regions with the highest number of cases.^9^ Therefore, it is necessary to use innovative approaches to improve the ability to recognize and detect leprosy cases early in the community, such as new diagnostic tests and screening questionnaires.^21,22^

Epidemiological risk of leprosy ranged between medium and high in the state of Goiás, however, in its macro-regions very low and low risk were predominant. For this reason, we decided to assess risk disaggregated by the municipalities of Goiás state as well. Thus, we found municipalities at high risk of leprosy, even though they belonged to macro-regions with low epidemiological risk.

These data are similar those found by research conducted in the Brazilian states of Minas Gerais^12^ and Paraíba,^11^ which showed heterogeneous distribution of epidemiological risk of leprosy in their municipalities. Social, economic and health disparities in territories act as factors of vulnerability to greater occurrence of leprosy,^23^ which favors heterogeneous regional behavior. Contextual characteristics, such as primary health care (PHC) coverage or access to health facilities, also contribute to the occurrence of disparities in the distribution of epidemiological risk of leprosy.^24,25^ Risk is multifactorial, since it involves the presence of determinants that are individual, social, economic, programmatic and political, as well as determinants of access to health services.^11,12^

Brazil has adopted decentralization of leprosy control actions to PHC services as a health policy. However, the epidemiological situation observed in Brazil and its regions reveals that PHC is still insufficient for leprosy to be controlled,^24^ whereby it is essential to reflect on its responsibilities and achievement of the goals proposed by the Ministry of Health.^4^

The increase in PHC service coverage has had an impact reducing endemic leprosy, however, this currently depends on the quality of the actions undertaken by health professionals, as well as prioritization by health service managers.^25^ Especially in the state of Goiás, PHC coverage does not appear to explain the different epidemiological risks observed, as, in general, coverage is greater than 90% in the state’s municipalities.^26^

The Global Leprosy Strategy 2021-2030: “Towards Zero Leprosy” aims to eliminate leprosy (defined as interruption of transmission), however, the strategy requires specific health service funding, sometimes through joint funding by partner organizations.^27^ In view of this new strategy, health policies need to incorporate several strategic approaches for macro-regions with high, medium and low epidemiological risk of leprosy in order to achieve the goal of eliminating it. 

We observed a decrease in the number of high-risk municipalities in the period 2019-2021, compared to the other three-year periods we assessed. As the leprosy epidemiological risk index is a composite indicator and refers to three epidemiological indicators, it may have been impacted by the covid-19 pandemic.^5^ World Health Organization data^1^ demonstrated a reduction in the detection of new leprosy cases at a faster rate than expected during the pandemic, while now, in the post-pandemic period, new leprosy cases have begun to increase again. However, in Goiás, despite the reduction in cases in 2019-2020, there was an increase in the epidemiological risk of leprosy (0.53-0.64), probably influenced by late detection, as expressed by the grade 2 physical disability indicator. 

Actions aiming at the control the covid-19 pandemic had a major impact on health service program activities intended to address diseases, especially in primary care. There was an impact on community actions for early detection of new leprosy cases, which resulted in late diagnosis and an increase in cases diagnosed with grades of physical disability.^5,28^ Factors such as cancellation of community activities^5^ and reduced health care, caused by restrictions in accessing health services or the population’s fear of seeking them, culminated in situations of instability in the programmatic control of chronic conditions and increased morbidity and mortality.^28^

National, state and municipal leprosy control programs must prioritize training and qualification of health professionals in order to avoid diagnostic errors and reduce delays in detection; sustainability of leprosy control in integrated health services must be guaranteed.^22^ Equitable training provision throughout the territory must also be guaranteed, regardless of epidemiological risk, as the differences observed in this study may be related to sensitivity regarding carrying out diagnosis, treatment, prevention of physical disabilities and contact surveillance (important for breaking the disease transmission chain), mainly by medical professionals, which may have reflected in the epidemiological situation of the Goiás macro-regions. 

We found differences in the trend which ranged between stationary in the state as a whole and falling in its Central-North, Northeast and Southwest macro-regions. Studies that assessed the trend of separate indicators in Goiás, in the pre-pandemic period, showed a falling trend in the general detection of new leprosy cases.^9,14^ Different dynamics have been observed in the proportion of cases with grade 2 physical disability, which has shown a growing trend in published research.^9,11^ The leprosy epidemiological risk index seems to be ideal for evaluating the leprosy burden in the territory, as it works from the perspective of a composite indicator. 

The trends we found need to be assessed with caution, as detection of cases with grade 2 physical disability (as observed in the separate indicators) reveals late diagnosis, making it necessary to strengthen public policies to address leprosy through health education, with use of information technology, communication and provision of training to health professionals in an equitable manner throughout the territory.

In conclusion, there was a stationary trend in the leprosy epidemiological risk index for the state of Goiás and two of its health macro-regions (Central-West and Central-Southeast) between 2010 and 2021. Three macro-regions showed a falling trend (Central-North, Northeast and Southwest). The percentage of municipalities with high epidemiological risk fell in the period. Despite the reduction, in 2021, the state still presented medium epidemiological risk of leprosy. Actions to reduce epidemiological risk of leprosy in Goiás are necessary, especially in macro-regions where the trend is stationary, including new measures for early diagnostic screening, treatment and prevention of disabilities, in addition to health education. Future studies assessing factors associated with epidemiological risk in Goiás and other Brazilian states are equally relevant for planning effective public policies.
