# Supplementary material for: Trend in the epidemiological risk of leprosy in the state of Goiás-Brazil between 2010 and 2021
Source: Epidemiol Serv Saude. 2024 Aug 23;33:e20231435. doi: 10.1590/S2237-96222024v33e20231435.en (PMC11346681; doi:10.1590/S2237-96222024v33e20231435.en)
Supplement: Translation

# Translation: Tendência do risco epidemiológico da hanseníase no estado de Goiás entre 2010 e 2021

**DOI:** 10.1590/S2237-96222024v33e20231435.pt

**Authors:** Caio Cesar Barbosa, Rafael Alves Guimarães, Nayara Figueiredo Vieira

**Affiliations:** 1 Universidade Federal de Goiás, Faculdade de Enfermagem, Goiânia, GO, Brasil

**Keywords:** Estudos de Séries Temporais, Epidemiologia, Monitoramento Epidemiológico, Vigilância em Saúde, Hanseníase

## Abstract

**Objetivo:**

Analisar a tendência do risco epidemiológico da hanseníase no estado de Goiás e macrorregiões de saúde de 2010 a 2021.

**Método:**

Análise de série temporal do indicador composto do índice composto de risco epidemiológico de hanseníase em Goiás. Utilizaram-se casos do Sistema de Informação de Agravos de Notificação para cálculo dos indicadores isolados e o risco foi classificado em alto, médio, baixo e muito baixo. A tendência foi analisada pela regressão linear de Prais-Winsten e foram produzidos mapas de risco.

**Resultados:**

Goiás apresentou alta endemicidade para hanseníase (24,8 casos/100 mil habitantes) e médio risco epidemiológico 2019 e 2021 (0,58). Observou-se tendência estacionária (variação percentual anual, 0,50; intervalo de confiança de 95%, -3,04; 4,16) para o risco da hanseníase em Goiás e macrorregiões Centro-Oeste e Centro-Sudeste.

**Conclusão:**

Verifica-se a necessidade de ações para reduzir o risco epidemiológico da hanseníase, especialmente com tendência estacionária, e isso inclui medidas de rastreamento precoce de novos casos e educação em saúde.

## Contribuições do estudo

**Principais resultados:**

A hanseníase persiste em Goiás, Brasil, de forma endêmica, com distribuição heterogênea. Houve redução do número de municípios com alto risco epidemiológico da hanseníase, porém os desafios incluem a transmissão ativa e o diagnóstico tardio.

**Implicações para os serviços:**

Estratégias de longo prazo de prevenção, detecção precoce, tratamento e acompanhamento de pacientes com hanseníase e seus contatos faz-se necessária.

**Perspectivas:**

Torna-se crucial fortalecer as políticas de saúde dirigidas à hanseníase no estado, priorizando programas de educação permanente e de formação para profissionais que atuam em todo o território.

## INTRODUÇÃO

A hanseníase permanece um problema de saúde em diversos países, incluindo o Brasil.^1,2^ Estima-se que em 2022 ocorreram 174.087 casos novos da doença, com aumento de 23,8% em relação a 2021.^1^

O Brasil ocupa o segundo lugar mundialmente em novos casos de hanseníase em termos absolutos.^1^ A taxa de detecção de novos casos de hanseníase no país foi de 8,58 casos/100 mil habitantes, indicando média endemicidade,^3^ e necessidade de implementar ações para atingir as metas estabelecidas pela Estratégia Nacional para Enfrentamento à Hanseníase 2024-2030, do Ministério da Saúde.^4^

A taxa de detecção de casos de hanseníase apresentou tendência decrescente no Brasil de 2011 a 2022, principalmente devido à poliquimioterapia e às ações estratégicas do Sistema Único de Saúde (SUS), como a descentralização das ações de controle da hanseníase para atenção primária à saúde. Contudo, verificou-se redução acelerada, superior ao esperado, no período pandêmico, entre os anos 2020 e 2022.^3^ As medidas de saúde pública adotadas para covid-19, como isolamento e restrições à circulação, dificultaram as ações de base populacional necessárias para a detecção precoce de novos casos, como busca ativa, exames de contato e outros; além disso, houve redução do acesso aos serviços de hanseníase no mesmo período.^5^

Estudos conduzidos no Brasil e na China^6-10^ avaliaram tendência temporal de indicadores isolados, sendo relevante avaliar o risco epidemiológico de maneira abrangente. Por meio do indicador composto de risco epidemiológico da hanseníase, os estados da Paraíba^11^ e Minas Gerais^12^ analisaram a distribuição espacial do risco, sendo constatada distribuição desigual do risco epidemiológico da hanseníase nos municípios que constituem os estados pesquisados.

O estado de Goiás destaca-se por altas taxas de detecção de hanseníase do Brasil e com áreas de risco para a doença no país.^3^ Observou-se tendência de queda na detecção de novos casos de 2007 a 2017, padrão similar ao encontrado em outras regiões. Altas proporções de incapacidade física^13^ e casos em crianças traduzem a realização de diagnóstico tardio e transmissão recente da doença, o que reforça a necessidade de estratégias coordenadas para reduzir o impacto da doença e de estudos sobre a distribuição de casos.^13,14^

A análise da dinâmica da distribuição espacial de agravos e doenças e a tendência do risco epidemiológico, em especial da hanseníase, em regiões endêmicas, como é o caso do estado de Goiás, se faz benéfica para a identificação do risco conforme o espaço geográfico. Além disso, este estudo pode contribuir para monitorar a efetividade das ações da Estratégia Nacional para Enfrentamento à Hanseníase 2024-2030.^4^ Dessa forma, o objetivo deste estudo foi analisar a tendência do risco epidemiológico da hanseníase no estado de Goiás e macrorregiões de saúde de 2010 e 2021. 

## MÉTODOS

Trata-se de estudo de séries temporais sobre as tendências do risco epidemiológico da hanseníase em Goiás de 2010 a 2021. 

O estudo foi realizado com dados de 1º de janeiro de 2010 a 31 de dezembro de 2021. A unidade de análise foi o estado de Goiás e suas cinco macrorregiões de saúde, a saber: Centro-Norte, Centro-Oeste, Centro-Sudeste, Nordeste e Sudoeste. O estado localiza-se na região Centro-Oeste do Brasil e apresenta importância epidemiológica no cenário nacional. 

A população foi composta por novos casos de hanseníase de residentes do estado de Goiás notificados de 2010 a 2021. 

Os dados foram extraídos do Sistema de Informação de Agravos de Notificação (Sinan) e disponibilizados pela Secretaria Estadual de Saúde de Goiás. Os casos foram filtrados segundo o modo de entrada “casos novos”, tendo sido excluídos aqueles com desfecho registrado como “erro diagnóstico”. 

Para a construção do indicador composto do índice composto de risco epidemiológico de hanseníase em Goiás, utilizaram-se a taxa anual de detecção de casos novos de hanseníase por 100 mil habitantes, taxa anual de detecção de novos casos de hanseníase na população de até 14 anos por 100 mil habitantes e a taxa de casos novos de hanseníase com grau 2 de incapacidade física no momento do diagnóstico. A metodologia foi conduzida seguindo as recomendações do Ministério da Saúde do Brasil.^15^

Após o cálculo dos indicadores supracitados, por ano e município, foi aplicada equação matemática para obter razão entre o valor observado (média dos valores registrados anualmente) e o máximo (maior valor observado nos anos avaliados),^11^ por município, macrorregiões e estado. Em seguida, para cálculo do índice de risco epidemiológico de hanseníase, os três indicadores foram somados e divididos por três. Os valores médios variaram entre 0 e 1, sendo “melhor” o menor valor (0) e “pior” o maior valor (1).

Os mapas de risco foram construídos utilizando-se o indicador composto do índice de risco epidemiológico de hanseníase por município e segundo triênio da série temporal. Conforme fundamentação de estudos anteriores,^11,12^ a classificação dos valores foi baseada nos quartis de distribuição e assim classificados: muito baixo risco (< 0,2), baixo risco (0,21-0,4), médio risco (0,41-0,6) e alto risco (> 0,6). Posteriormente, mapas coropléticos foram criados por meio do *software* RStudio, na versão 1.4.1106.

As análises de tendências temporais foram realizadas no *software* estatístico Stata, versão 17.0 (StataCorp LP, College Station, Estados Unidos). 

Foi utilizado o modelo de regressão linear de Prais-Winsten, com variância robusta e ajuste para autocorrelação de Durbin-Watson, para analisar tendência temporal do índice de risco epidemiológico da hanseníase no estado de Goiás e suas macrorregiões, entre 2010 e 2021.^16^

A variável dependente (Y) do estudo foi o índice do risco epidemiológico da hanseníase e a variável independente foi o ano da série temporal. Antes da inclusão nos modelos de regressão, foi realizada a transformação em logaritmo de base 10 da variável dependente (Y). Esse procedimento possibilitou minimizar a heterogeneidade das variâncias dos resíduos do modelo de regressão e contribuiu para melhor apuração da tendência temporal.^16^ A equação de regressão de Prais-Winsten foi a seguinte:


$$Log(Y_{t})=\beta_{0}+\;\beta_{1}x\;+\;e_{t},$$

em que: *Log*(*Y* *t*) foi a variável dependente do estudo, ou seja, o índice do risco epidemiológico da hanseníase após a transformação em logaritmo de base 10, *β* 0 foi a constante ou intercepto; *β*_1_ foi a inclinação da reta ou coeficiente de tendência linear; x foi a variável independente, ou seja, o ano do estudo e *e* *t* o erro aleatório. O subscrito “t” estimou os tempos do conjunto de dados {t_1_, ..., t_12_}, no caso t_1_ = 2010 e t_19_ = 2021.Os coeficientes *β*_1_ e erros-padrões robustos (EPR) foram obtidos na análise de regressão de Prais-Winsten, e calculada a variação percentual anual (VPA) e o intervalo de confiança de 95% (IC_95_%) por meio das fórmulas:^16^


$$VPA=(1\;+\;10{)}^{\beta_{1}}*100,$$

sendo *β* 1 a inclinação da reta ou o coeficiente de tendência linear. 


$$IC95\%=(1\;+\;10^{\left(\beta_{1}\pm t*EPR\right)})*100,$$

*β* 1 é a inclinação da reta ou o coeficiente de tendência linear; *t* é o valor da distribuição *t* de *Student* e apresenta 11 graus de liberdade (12 anos de séries temporais – 1) a um IC_95_% bicaudal; e EPR é o erro-padrão robusto. 

A estatística *t* foi usada para estabelecer a significância estatística dos modelos, e valores de p < 0,05 foram considerados estatisticamente significativos. Com base nos resultados da VPA e valores de p obtidos nos modelos de regressão, as tendências foram classificadas em crescentes (VPA positiva e valor de p significativo), decrescentes (VPA negativa e valor de p significativo) ou estacionárias (VPA positiva ou negativa e p-valor não significativo). O coeficiente de determinação (*R* ^2^) foi usado para verificar o ajuste do modelo de regressão.

A pesquisa foi aprovada pelo Comitê de Ética em Pesquisa do Hospital das Clínicas da Universidade Federal de Goiás, sob Certificado de Apresentação de Apreciação Ética 58175122.3.0000.5078 e parecer nº 5.421.173, de 20 de maio de 2022.

## RESULTADOS

Entre 2010 e 2021, foram notificados 19.728 casos novos de hanseníase no estado de Goiás, sendo 814 (4,13%) em menores de 15 anos e 1.264 (6,40%) casos com grau 2 de incapacidade física no diagnóstico. 

A Tabela 1 apresenta as médias dos indicadores utilizados para o cálculo do indicador composto, segundo macrorregiões de Goiás. As médias anuais das taxas de detecção anual de casos novos de hanseníase, de detecção anual de novos casos de hanseníase na população de até 14 anos e de casos novos de hanseníase com grau 2 de incapacidade física no diagnóstico em Goiás, entre 2010 e 2021, foram de 24,8, 4,5 e 1,5 casos por 100 mil habitantes, respectivamente. As macrorregiões Centro-Oeste, Centro-Norte e Centro-Sudeste apresentaram taxa de detecção de casos novos de hanseníase acima do valor médio do estado (Centro-Oeste: 39,0 casos/100 mil habitantes, Centro-Norte: 25,1 casos/100 mil habitantes e Centro-Sudeste: 29,2 casos/100 mil habitantes). De forma similar, essas macrorregiões apresentaram taxa de detecção de casos novos de hanseníase na população de até 14 anos maior que a média estadual (Centro-Oeste: 7,0 casos/100 mil habitantes, Centro-Norte: 4,3 casos/100 mil habitantes e Centro-Sudeste: 6,2 casos/100 mil habitantes). Por fim, três regiões também apresentaram taxa de casos novos de hanseníase com grau 2 de incapacidade física no diagnóstico maior que a média do estado (Centro-Oeste: 2,6 casos/100 mil habitantes, Centro-Sudeste: 2,0 casos/100 mil habitantes e Sudoeste: 1,6 caso/100 mil habitantes).

**Tabela 1:** Taxa de detecção anual de casos novos de hanseníase na população geral e de até 14 anos e taxa de casos novos de hanseníase com grau 2 de incapacidade física no diagnóstico em Goiás, de 2010 a 2021 (n = 19.728)

Unidade de análise	Taxa de detecção anual de casos novos de hanseníase	Taxa de detecção anual de casos novos de hanseníase, na população de até 14 anos	Taxa de casos novos de hanseníase com grau 2 de incapacidade física no momento do diagnóstico
Centro-Norte	39,0	7,0	2,6
Centro-Oeste	25,1	4,3	1,1
Centro-Sudeste	29,2	6,2	2,0
Nordeste	11,4	2,6	0,9
Sudoeste	20,5	2,4	1,6
Goiás	24,8	4,5	1,5

Em Goiás, verificou-se incremento de 1,72% no índice de risco epidemiológico de hanseníase de 2010 para 2021 (0,58 para 0,59) (Figura 1). Os percentuais de mudança foram de -45,45% no Centro-Norte (0,11 para 0,06), -33,33% no Centro-Oeste (0,12 para 0,08), -33,33% no Centro-Sudeste (0,12 para 0,08), -50,00% no Nordeste (0,16 para 0,08) e -43,75% no Sudoeste (0,16 para 0,09). 

**Figura 1:** Índice de risco epidemiológico de hanseníase por macrorregião de Goiás, de 2010 a 2021 (n = 19.728)

O percentual de municípios com classificação de risco “muito baixo” variou de 24,4% para 45,1% entre os triênios 2010/2012 e 2019/2021, incremento de 84,83%. Por outro lado, o percentual de municípios classificados como “alto risco” variou de 11,0% para 3,7% entre os triênios 2010/2012 e 2019/2021, redução de 66,36%.

A Figura 2 apresenta a distribuição do risco epidemiológico da hanseníase nos municípios por triênio do estudo. As macrorregiões de saúde Centro-Norte, Centro-Oeste e Centro-Sudeste foram as que registraram maior número de municípios classificados como de alto risco epidemiológico da hanseníase.

**Figura 2:** Distribuição espacial por triênio do risco epidemiológico da hanseníase, por município do estado de Goiás, de 2010 a 2021 (n = 246)

**Tabela 2:** Evolução da classificação de risco epidemiológico da hanseníase dos municípios de Goiás por triênio de avaliação, de 2010 a 2021 (n = 246)

Classificação de risco	**2010-2012**		2013-2015		2016-2018		2019-2021	
	N	%	N	%	N	%	N	%
Muito baixo	60	24,4	74	30,1	91	37,0	111	45,1
Baixo	115	46,7	109	44,3	107	43,5	103	41,9
Médio	44	17,9	41	16,7	38	15,4	23	9,3
Alto	27	11,0	22	8,9	10	4,1	9	3,7

O índice do risco epidemiológico da hanseníase no estado de Goiás apresentou tendência estacionária no período em estudo (VPA = 0,50; IC_95_% -3,04;4,16). A tendência estacionária também foi observada nas macrorregiões Centro-Oeste (VPA = -4,04; IC_95_% -12,69;5,46) e Centro-Sudeste (VPA = 0,17; IC_95_% -13,21;16,57). Por outro lado, tendência decrescente foi observada nas macrorregiões Centro-Norte (VPA = -17,69; IC_95_% -24,40;-10,38), Nordeste (VPA = -9,17; IC_95_% -13,21;-6,18) e Sudoeste (VPA = -11,82; IC_95_% -18,01;-5,17) (Tabela 3).

**Tabela 3:** Variação percentual anual (VPA), intervalo de confiança de 95% (IC_95_) e coeficiente de determinação (R²) do índice do risco epidemiológico da hanseníase, por macrorregião do estado de Goiás, de 2010 a 2021, Goiás

Unidade de análise	VPA^a^ (IC_95_ **%**)	p-valor	R^2^ ^b^	Tendência
Centro-Norte	-17,69 (-24,40;-10,38)	0,002	0,668	Decrescente
Centro-Oeste	-4,04 (-12,69;5,46)	0,359	0,001	Estacionária
Centro-Sudeste	0,17 (-8,70;16,57)	0,969	0,001	Estacionária
Nordeste	-9,17 (-13,21;-6,18)	< 0,001	0,953	Decrescente
Sudoeste	-11,82 (-18,01;-5,17)	0,003	0,802	Decrescente
Goiás	0,50 (-3,04;4,16)	0,768	0,084	Estacionária

a) VPA: Variação percentual anual; b) Regressão linear de Prais-Winsten; R^2^: Coeficiente de determinação.

## DISCUSSÃO

Os resultados deste estudo indicam que a hanseníase persiste como problema de saúde pública no estado de Goiás. As médias anuais das taxas de detecção anual de casos novos de hanseníase, de detecção anual de novos casos de hanseníase na população de até 14 anos e de casos novos de hanseníase com grau 2 de incapacidade foram altas, especialmente nas macrorregiões Centro-Oeste, Centro-Norte e Centro-Sudeste. Isso sugere uma endemicidade significativa, transmissão recente com presença de casos em crianças e realização do diagnóstico tardio da hanseníase. Verificou-se que o percentual de municípios com alto risco para hanseníase se reduziu em Goiás de 2010/2021 para 2019/2021. No entanto, observaram-se distribuições heterogêneas no número de municípios com alto risco para hanseníase. As macrorregiões de saúde Centro-Norte, Centro-Oeste e Centro-Sudeste foram as que registraram maior número de municípios classificados como de alto risco epidemiológico da hanseníase. Por fim, observou-se uma tendência estacionária para o risco epidemiológico da hanseníase no estado de Goiás e tendência decrescente para três macrorregiões (Centro-Norte, Nordeste e Sudoeste).

Este estudo utilizou fontes de dados secundários, que podem conter inconsistências na qualidade das informações. Para minimizar vieses ou erros, os dados foram tratados com rigor ético e metodológico. No último triênio avaliado (2019 e 2021), em função da covid-19, pode ter havido casos não diagnosticados de hanseníase e, assim, subestimação da situação epidemiológica da doença. Dessa forma, recomendam-se novos estudos sobre o risco epidemiológico da hanseníase após 2020 para se confirmar a situação da endemia nas macrorregiões. 

A hanseníase persiste como problema no território goiano, em especial na macrorregião Centro-Norte, com alta endemicidade para a doença e realização de diagnóstico tardio. Essa macrorregião é historicamente marcada por maior número de casos no estado de Goiás, devido a sua localização limítrofe com estados de alta endemicidade, como Mato Grosso e Tocantins.^3^ Assim, o processo migratório pode explicar a disparidade da macrorregião Centro-Norte em relação ao estado de Goiás. Porém, a doença pode se associar a outros fatores, como maior vulnerabilidade ambiental ou individual.^17^

Com média de número de casos de 2009 e 2022 de 1.685 casos ao ano,^3^ o estado de Goiás está longe da meta de eliminação proposta pela Estratégia 2024-2030 do Ministério da Saúde, que prevê eliminar a hanseníase pela ausência de casos autóctones pelo período de três anos consecutivos após interrupção da transmissão.^4^ Apesar disso, o estado segue tendência de queda na detecção de novos casos de hanseníase, mesmo panorama observado mundialmente.^1,11,14^ Acredita-se que o número absoluto de novos casos de hanseníase, proposto pela estratégia, não seja a melhor maneira de avaliar a interrupção da transmissão, mas, sim, um agregado de indicadores que permitam avaliar de forma robusta o estágio de eliminação da doença. 

Porém, dinâmica diferente foi observada na taxa de casos com grau 2 de incapacidade física, que mostraram tendência crescente nas pesquisas.^9,13^ A persistência de casos diagnosticados com incapacidade física grau 2 em níveis elevados evidencia diagnóstico tardio e contribui para a formação de focos de transmissão da doença.^11,18^ Estudo realizado na Etiópia evidenciou atraso médio de 12 meses no diagnóstico, que culminou em 83% de incapacidade física 2 nos pacientes avaliados.^19^

Três pilares são necessários para a detecção precoce: educação em saúde para a população, capacitação de profissionais de saúde e investimento em políticas de saúde que atendam às necessidades da população. Oferecer educação em saúde às comunidades é vital para apoiar o reconhecimento precoce dos sintomas da hanseníase e reduzir o preconceito e a estigmatização dos pacientes com hanseníase e suas famílias;^19^ esses fatores podem contribuir para reduzir o atraso no diagnóstico e prevenir as incapacidades físicas.

Desde 2019, o número de casos de hanseníase em menores de 15 anos vem diminuindo no estado de Goiás, no entanto, média de 67 casos de crianças ao ano foi observada de 2009 a 2022.^20^ Isso demonstra transmissão recente da doença, e também que é preciso refletir sobre estratégias que alcancem a meta atual do Ministério da Saúde, que prevê ausência de casos autóctones em pessoas com menos de 15 anos pelo período de cinco anos consecutivos.^4^ Estudo realizado na China observou tendência decrescente de novos casos infantis de hanseníase entre 2011 e 2020, porém agrupados em áreas de alto risco, que foram consideradas como regiões com maior número de casos.^9^ Assim, torna-se necessário utilizar abordagens inovadoras para melhorar a capacidade de reconhecer e detectar casos de hanseníase precocemente na comunidade, como novos testes diagnósticos e questionários de triagem.^21,22^

O risco epidemiológico de hanseníase oscilou entre médio e alto no estado de Goiás, entretanto, nas macrorregiões houve predominância de risco muito baixo e baixo. Por esse motivo, optou-se por também avaliar o risco desagregado pelos municípios goianos. Assim, foram observados municípios de alto risco para hanseníase, mesmo eles pertencendo a macrorregiões de baixo risco epidemiológico.

Esses dados assemelham-se a pesquisas realizadas nos estados de Minas Gerais^12^ e Paraíba,^11^ que mostraram distribuição heterogênea do risco epidemiológico da hanseníase nos municípios. As disparidades sociais, econômicas e de saúde nos territórios atuam como fatores de vulnerabilidade para maior ocorrência da hanseníase,^23^ o que favorece comportamento regional heterogêneo. As características contextuais, como cobertura de atenção primária a saúde (APS) ou acesso aos equipamentos de saúde, também contribuem para a ocorrência de disparidades na distribuição do risco epidemiológico da hanseníase.^24,25^ O risco é multifatorial, pois se vincula à presença de determinantes individuais, sociais, econômicos, programáticos, políticos e de acesso aos serviços de saúde.^11,12^

No Brasil, adotou-se como política de saúde a descentralização das ações de controle da hanseníase para os serviços de atenção primária. No entanto, a situação epidemiológica observada no país e em suas regiões revela que a APS ainda é insuficiente para o controle da doença,^24^ sendo fundamental a reflexão sobre suas atribuições para o alcance das metas propostas pelo Ministério da Saúde.^4^

O aumento da cobertura dos serviços de APS impactou na redução da endemia, no entanto, atualmente depende da qualidade das ações que são desenvolvidas pelos profissionais de saúde, bem como da priorização pelos gestores.^25^ Especialmente no estado de Goiás, a cobertura de APS parece não justificar os diferentes riscos epidemiológicos observados, pois, no geral, a cobertura é maior que 90% nos municípios goianos.^26^

A Estratégia Global de Hanseníase (2021-2030) “Rumo à zero hanseníase” tem como meta eliminar a hanseníase (definida como interrupção da transmissão), no entanto, ela deve ser incluída nos orçamentos para financiamento, às vezes complementado por contribuições de parceiros.^27^ Devido a essa nova estratégia, políticas de saúde devem incorporar diversas abordagens estratégicas para as macrorregiões com alto, médio e baixo risco epidemiológico da hanseníase e alcançar a meta de eliminação da doença. 

Constatou-se diminuição no número de municípios de alto risco em 2019-2021, em comparação aos outros triênios. Como o índice de risco epidemiológico de hanseníase é indicador composto e refere-se a três indicadores epidemiológicos, ele pode ter sido impactado pela pandemia da covid-19.^5^ Dados da Organização Mundial da Saúde^1^ demonstraram redução na detecção de novos casos de hanseníase com velocidade mais acelerada do que o esperado, e agora, no cenário pós-pandemia, há retomada no aumento de novos casos da doença. No entanto, no estado de Goiás, apesar da redução de casos em 2019-2020, houve aumento do risco epidemiológico da hanseníase (0,53-0,64), provavelmente influenciado pela detecção tardia, que é traduzido pelo indicador grau 2 de incapacidade física. 

As ações destinadas ao controle da pandemia impactaram sobremaneira nas atividades de enfrentamento dos programas realizadas pelos serviços, especialmente na atenção primária. Houve impacto em ações comunitárias de detecção precoce de novos casos de hanseníase, o que se traduziu em diagnóstico tardio e aumento de casos diagnosticados com grau de incapacidade física.^5,28^ Fatores como cancelamento de atividades comunitárias^5^ e desassistência, provocada pelas restrições de acesso ou por receio da população em buscar os serviços de saúde, culminaram em situações de instabilidade do controle programático das condições crônicas e aumentaram a sua morbimortalidade.^28^

Os programas nacionais, estaduais e municipais de controle da hanseníase devem priorizar o treinamento e a capacitação de profissionais de saúde para evitar erros de diagnóstico e reduzir atrasos na detecção; deve-se garantir a sustentabilidade do controle da hanseníase nos serviços integrados de saúde.^22^ Assegurar, também, a oferta equânime de treinamentos em todo território, independentemente do risco epidemiológico, visto que as diferenças observadas neste estudo podem estar relacionadas à sensibilidade de realizar diagnóstico, tratamento, prevenção de incapacidades físicas e vigilância de contatos (importante para a quebra da cadeia de transmissão da doença), principalmente pelos profissionais médicos, o que pode ter se refletido na situação epidemiológica das macrorregiões do estado de Goiás. 

Foram observadas diferenças, na tendência de risco epidemiológico da hanseníase, oscilando entre estacionária no estado e decrescente nas macrorregiões Centro-Norte, Nordeste e Sudoeste. Estudos que avaliaram a tendência de indicadores isolados no estado de Goiás no período pré-pandemia evidenciaram direção decrescente na detecção geral de novos casos de hanseníase.^9,14^ Dinâmica diferente foi observada na proporção de casos com grau 2 de incapacidade física, que mostraram tendência crescente nas pesquisas.^9,11^ O índice do risco epidemiológico da hanseníase parece ser o ideal para avaliar a carga da doença no território, pois trabalha com perspectiva de indicador composto. 

As tendências observadas devem ser avaliadas com cautela, pois a detecção de casos com grau 2 de incapacidade física (como observada nos indicadores isolados) revela diagnóstico tardio, sendo necessário fortalecer as políticas públicas de enfrentamento da hanseníase por meio da educação em saúde com o uso de tecnologia de informação, comunicação e oferta de capacitações a profissionais de saúde de maneira equânime no território.

Em conclusão, verificou-se uma tendência decrescente do índice do risco epidemiológico para o estado de Goiás e duas macrorregiões de saúde (Centro-Oeste e Centro-Sudeste) entre 2010 e 2021. Três macrorregiões apresentaram tendência decrescente (Centro-Norte, Nordeste e Sudoeste). O percentual de municípios com alto risco epidemiológico apresentou queda no período. Apesar da redução, em 2021, o estado ainda apresentou médio risco epidemiológico para hanseníase. Ações para redução do risco epidemiológico da hanseníase em Goiás se mostram necessárias, especialmente nas macrorregiões com tendência estacionária, incluindo-se novas medidas de rastreamento diagnóstico precoce, tratamento e prevenção de incapacidades, além de educação em saúde. Estudos futuros que avaliem fatores associados ao risco epidemiológico em Goiás e outros estados são igualmente relevantes para planejar políticas públicas eficazes.
